# Predictive assays for craniofacial malformations: evaluation in *Xenopus* *laevis* embryos exposed to triadimefon

**DOI:** 10.1007/s00204-022-03327-w

**Published:** 2022-06-24

**Authors:** Maria Battistoni, Francesca Metruccio, Francesca Di Renzo, Renato Bacchetta, Elena Menegola

**Affiliations:** 1 Department of Physics Aldo Pontremoli, Università Degli Studi Di Milano, via Celoria, 16-20133 Milan, Italy; 2 Department of Biomedical and Clinical Sciences, ICPS, ASST Fatebenefratelli Sacco, Università degli studi di Milano, Via GB Grassi, 74- 20159 Milan, Italy; 3 Department of Environmental Science and Policy, Università Degli Studi Di Milano, via Celoria, 26-20133 Milan, Italy

**Keywords:** Craniofacial defects, BMD approach, Alternative animal model, R-FETAX

## Abstract

Craniofacial defects are one of the most frequent abnormalities at birth, but their experimental evaluation in animal models requires complex procedures. The aim of the present work is the comparison of different methodologies to identify dose- and stage-related craniofacial malformations in *Xenopus* *laevis* assay (R-FETAX, where the full cartilage evaluation, including flat mount technique, is the gold standard for skeletal defect detection). Different methods (external morphological evaluation of fresh samples, deglutition test, whole mount cartilage evaluation and Meckel–palatoquadrate angle measurements) were applied. Triadimefon (FON) was selected as the causative molecule as it is known to induce craniofacial defects in different animal models, including the amphibian *X.* *laevis*.

FON exposure (0–31.25 μM) was scheduled to cover the whole 6-day test (from gastrula to free swimming tadpole stage) or each crucial developmental phases: gastrula, neurula, early morphogenesis, late morphogenesis, tadpole. Dose-dependent effects (fusions among craniofacial cartilages) were evident for groups exposed during the morphogenetic periods (neurula, early morphogenesis, late morphogenesis); gastrula was insensitive to the tested concentrations, tadpole group showed malformations only at 31.25 μM. The overall NOAEL was set at 3.9 μM. Results were evaluated applying benchmark dose (BMD) approach. The comparison of relative potencies from different methods showed deglutition as the only assay comparable with the gold standard (cartilage full evaluation).

In conclusion, we suggest deglutition test as a reliable method for a rapid screening of craniofacial abnormalities in the alternative model *X.* *laevis*. This is a rapid, inexpensive and vital test allowing to preserve samples for the application of further morphological or molecular investigations.

## Introduction

The group “Craniofacial congenital anomalies” is the most frequent category of malformations diagnosed at birth and comprises abnormalities of skull, jaws and related soft tissues. Usually, children with this kind of diagnosis require constant specific medical support along the years, involving pediatricians, surgeons, orthodontists, ophthalmologists, speech therapists and other specialists. The most frequent craniofacial defects diagnosed at birth (occurring in about 1:700 live births) are oral clefts in any form (cleft lip and/or palate alone or syndromic with other head skeletal defects or other anomalies) (Mossey et al. 2009). Other craniofacial anomalies include jaw deformities, malformed or missing teeth, defects in the ossification of facial or cranial bones, and facial asymmetries. The impairment of the normal craniofacial morphogenesis has been related to multifactorial causes, involving both genetic and environmental risk factors (Mossey et al. 2009) and is suggested in the process of syndrome diagnoses (Hallgrímsson et al. 2020).

The experimental evaluation of skeletal alteration is a not simple concern. In classical models (evaluation of mammal fetuses exposed in utero to xenobiotics), the external morphology is not adequate to delineate the majority of skeletal defects (including fusions among skeletal structures) and also the routine staining of the fetal skeleton (single stain for bones) fails to individuate most alterations. Consequently, a more complex procedure is needed (double staining for bone and cartilage) (Menegola et al. 2002). In particular, double staining of the craniofacial skeleton allowed the deep detection of complex pictures induced by the exposure of rodent embryos to the agricultural fungicide triadimefon (FON): the early exposure of mouse (E8, E9, E10, E11 or E12) or rat (E9.5, E10, E11 or E12) embryos produced typical severe craniofacial defects (agenesis or abnormal shape of craniofacial elements; fusions involving several jaw/ear/skull elements; ectopic upper jaw cartilage) in a stage-related manner (Menegola et al. 2005; Di Renzo et al. 2007, 2011c). We also described craniofacial defects related to FON exposure in the amphibian alternative model *Xenopus* *laevis*, where fusions between maxillary and mandibular cartilages induced a circular unarticulated funnel-shaped mouth (Groppelli et al. 2005; Papis et al. 2007; Di Renzo et al. 2011b, e).

Both in mammals and in *X.* *laevis*, FON-related pathogenic adverse outcome pathway supposes an imbalance in retinoic acid catabolism leading to abnormal hindbrain segmentation and abnormal neural crest cell specification and migration (Menegola et al. 2005; Groppelli et al. 2005; Papis et al. 2007; Di Renzo et al. 2007, 2011b, d). The full adverse outcome pathway has been described considering data obtained in different experimental models (Menegola et al. 2021).

To characterize FON effects on craniofacial morphogenesis, the use of zebrafish (*Danio* *rerio*) embryo model has also been proposed in alternative to the classical models (Zoupa and Machera 2017; Zoupa et al. 2020). The alterations were detected in fresh samples (showing hypolastic/ flattened brain vesicles) (Zoupa and Machera 2017) or through the measurement of the angle formed by the Meckel's and palatoquadrate stained cartilages (M-–PQ angle increased in a concentration-related manner, as described by Zoupa et al. (2020).

The aim of the present work is the identification of the most suitable test predictive for craniofacial malformation assessment in *X.* *laevis*. FON was selected as causative molecule, due to its known properties in inducing craniofacial defects in *X.* *laevis* (Groppelli et al. 2005; Papis et al. 2007; Di Renzo et al. 2011b, e). The full cartilage evaluation was selected as gold standard for detailed skeletal defect evaluation (Pasqualetti et al. 2000; Spokony et al. 2002; Baltzinger et al. 2005; Dubey and Saint-Jeannet 2017). Different methods (external morphological evaluation of fresh samples, deglutition test, whole mount evaluation of cartilages and M–PQ angle measurements) were considered to select the most predictive one for craniofacial abnormality detection. The previously described windowed approach (R-FETAX, Battistoni et al. 2022) was applied.

## Materials and methods

### R-FETAX

**Fig.1:**
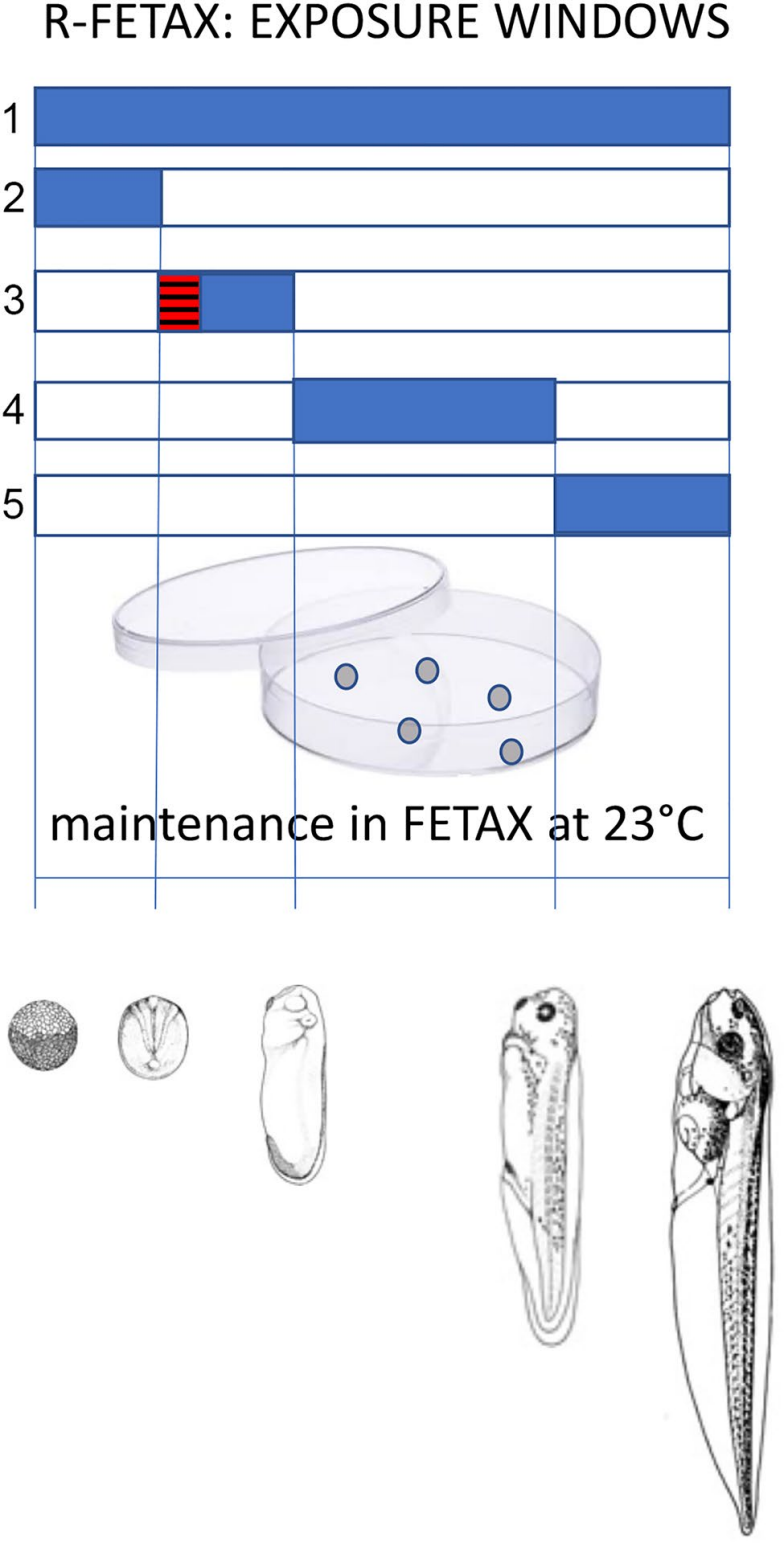
R-FETAX experimental approach. The classical FETAX exposure (1) covers the entire test. R-FETAX windowed approach, by contrast, provides limited times of exposure, corresponding to the main developmental phases: 2 = pre-organogenetic period (midblastula-gastrula); 3 = early organogenetic period (from neurula till phylotypic stages; the striped red area corresponds to neurula); 4 = late organogenetic period (from phylotipic stages till tadpole); 5 = tadpole (very late organogenesis and functional differentiation)

All reagents were purchased from Sigma, Italy. Amphibian *X.* *laevis* adults (Nasco, USA) were maintained in an automatic breeding system (Tecnoplus, Techniplast, Italy) under controlled water conditions (*T* = 20 ± 2 °C; pH = 7.5 ± 0.5; conductivity = 1000 ± 100 μS), 12-h light/dark cycle (light from 7:00 AM to 7:00 PM) and fed with a semisynthetic diet twice a week (XE40 by Mucedola; Settimo Milanese, Italy). Embryos were obtained as described by Battistoni et al. (2022) without adult human chorionic gonadotropin injection. The collected embryos were cleaned by gentle swirling in a 2.25% L-cysteine solution with an arranged pH of 8.0 and rinsed several times in FETAX solution, whose composition was 625 mg/L NaCl, 96 mg/L NaHCO_3_, 30 mg/L KCl, 15 mg/L CaCl_2_, 60 mg/L CaSO_4_ ·2H_2_O, and 70 mg/L MgSO_4_. To obtain the test solutions, FON was properly dissolved in 100% ethanol and diluted in FETAX solution (2.5 μl/mL) to obtain the final concentration of 0–3.9–7.8–15.625–31.25 μM FON. Normally cleaved embryos were selected for testing and specific stages evaluated according to Nieuwkoop and Faber (1956). During the whole test time (6 days, considering 0 the morning after egg deposition), samples were maintained in a thermostatically controlled FETAX solution (5 embryos/5 mL, in Petri dishes at 23 ± 0.5 °C) from NF stage 8 until day 6, corresponding to NF stage 46, as evaluated in preliminary tests on unexposed larvae. Exposures covered the whole length of the standard FETAX procedure (NF stage 8–46, classical FETAX exposure group) or was limited to windows covering some developmental phases considered of interest: *i*) from day 0 to day 0.5 (NF stage 8–13, gastrula); *ii*) from day 0.5 to day 1 (NF stage 13–26, early morphogenesis); *iii*) from day 1 to day 2 (NF stage 26–38, late morphogenesis); i*v*) from day 2 to day 6 (NF stages 38–46; tadpole stages). An extra group was exposed during the same window previously used in our publications on effects of FON in *X.* *laevis* (from day 0.5 to day 0.75, NF stage 13–17, neurula) (Groppelli et al. 2005; Papis et al. 2007; Di Renzo et al. 2011a, b, e) (Fig. 1).

### Deglutition test and gross morphological evaluation

**Fig.2:**
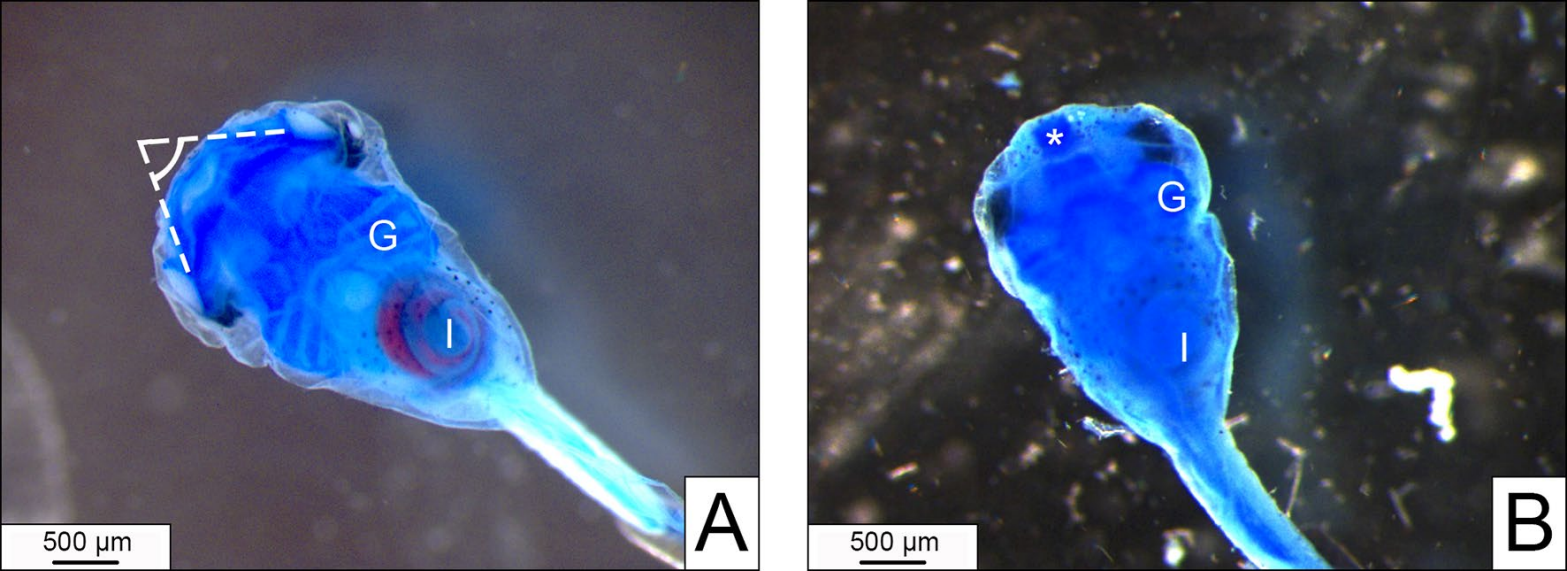
Whole mount alcian blue-stained craniofacial cartilages (ventral view), allowing to detect cartilaginous elements in a normal larva **(A)** and in an abnormal sample **(B)** characterized by circular funnel-shaped fused cartilages (*). G = gill basket; I = intestine with degl + **(A)** and degl− **(B)** phenotypes (respectively, presence/absence of red microplastics in the intestine). In A, dotted lines indicate the M–PQ angle measurement

At day 6, the deglutition test was performed as previously described by Battistoni et al. (2022), with some modifications: larvae were maintained for 2 h at 23 ± 0.5 °C in FETAX solution containing 25 μg/mL red polystyrene microparticles (1 µm diameter, Sigma). Larvae, overdosed at 4 °C with anesthetic (0.02% MS222, dissolved in FETAX solution), were evaluated under a dissecting microscope (Leica) for gross morphology and presence of microplastics in the intestine and photographed. The deglutition test is an indirect evaluation of jaw functionality because only larvae with functioning jaws can ingest microplastic. The test was considered negative (degl−) in tadpoles with no red color in the intestine (Fig. 2). In doubt cases, fixed tadpoles were reevaluated during cartilage evaluation by excision of the abdominal wall and the deep evaluation of the intestinal spires. Euthanized larvae were rinsed in FETAX, fixed in ethanol 50%, conserved in ethanol 70% and processed for cartilage staining.

### Cartilage staining and flat mount procedure

Tadpoles were processed for whole mount cartilage staining as previously described (Di Renzo et al. 2011a). The blue staining solution was composed of 0.02% alcian blue in ethanol 70% containing 40 mM MgCl_2_. Samples were incubated at RT under stirring overnight in the staining solution, rinsed in ethanol 70%, and observed under a dissecting microscope (Leica). Cartilages appeared dark blue, and connectives light blue. Whole mount stained cartilages were morphologically evaluated and photographed. The flat mount technique (Di Renzo et al. 2011a) was applied to evaluate the mouth articular regions in detail.

### M–PQ angle measurement

The ventral view picture of alcian blue-stained craniofacial cartilages was taken to measure the M–PQ angle according to Zoupa et al. (2020), adapted to *X.* *laevis* tadpole (Fig. 2A). The ImageJ software was used to take the M–PQ angle measure.

### Statistical analysis and mathematical data modeling

Continuous data, expressed as mean and standard deviation, were analyzed using ANOVA followed by Tukey’s post hoc test. Frequencies were analyzed using the Chi-square for trend test. The level of significance was set at *p* < 0.05.

The benchmark dose (BMD) approach was applied using PROAST (67 version), a software package developed by the Dutch National Institute for Public Health and the Environment (RIVM) (www. proast.nl) for the statistical analysis and modeling of dose–response toxicological data.

Cartilage evaluation in detail (flat mount) was selected as the gold standard method to detect facial abnormalities and articular defects. Data were modeled and dose–response curves obtained, setting the benchmark dose (BMD) at 10% benchmark response (BMR), considering stage as covariate.

On the basis of the covariate results, Hill model indicated the group exposed during the late morphogenetic period as the sensible subgroup and subsequent analyses were performed on data obtained in this group. Thereafter, we modeled results obtained with the different methodologies (external gross morphology, deglutition test, whole mount alone or combined with flat mount cartilage evaluation) and BMDs at 10% benchmark response (BMR) were derived.

Finally, for every developmental window exposed group, the exponential model family equations were selected to describe the dose–response curves and obtain the relative potency factors (RPFs) of deglutition test versus the gold standard method (full evaluation of cartilage).

## Results

### Dose- and stage-dependent effects of FON

No embryo-lethal effects were observed in groups exposed to FON during the whole test or the different developmental windows. The full evaluation of stained cartilages revealed, at the tested concentrations, no effects during the pre-organogenetic period (gastrulation). On the contrary, specific dose-related effects on craniofacial morphogenesis were observed after FON exposure during the classical FETAX period (whole test) and during the organogenetic periods (neurula, early/late morphogenetic windows and tadpole window) (Table 1).

**Table 1:** Percentage of larvae with abnormal cartilages in groups exposed to FON at different developmental stages

Exposure window	Exposure NF stages	Larvae with cartilage abnormalities	Fon concentration (μm)					*p* value
			**0**	**3.9**	**7.8**	**15.6**	**31.2**	
Classical FETAX exposure	**8–46**	Abnormal (full evaluation)	**0**	**0**	**100**	**100**	**100**	** < 0.0001**
		Fused (flat mount)	0	0	27	0	0	
		Fused (whole mount)	0	0	73	100	100	
Gastrula	**8–13**	Abnormal (full evaluation**)**	**0**	**0**	**0**	**0**	**0**	> 0.05
		Fused (flat mount)	0	0	0	0	0	
		Fused (whole mount)	0	0	0	0	0	
Neurula	**13–17**	Abnormal (full evaluation)	**0**	**0**	**0**	**13**	**93**	** < 0.0001**
		Fused (flat mount)	0	0	0	13	40	
		Fused (whole mount)	0	0	0	0	53	
Early morphogenesis	**13–26**	Abnormal (full evaluation)	**0**	**0**	**93**	**100**	**100**	** < 0.0001**
		Fused (flat mount)	0	0	80	0	0	
		Fused (whole mount)	0	0	13	100	100	
Late morphogenesis	**26–38**	Abnormal (full evaluation)	**0**	**7**	**86**	**100**	**93**	** < 0.0001**
		Fused (flat mount)	0	7	86	100	87	
		Fused (whole mount)	0	0	0	0	6	
Tadpole	**38–46**	Abnormal (full evaluation)	**0**	**0**	**0**	**0**	**29**	**0.0061**
		Fused (flat mount)	0	0	0	0	29	
		Fused (whole mount)	0	0	0	0	0	

Bold characters in *p* value column are for significant values

Statistics by Chi-square for trend calculated on frequencies

**Fig. 3:**
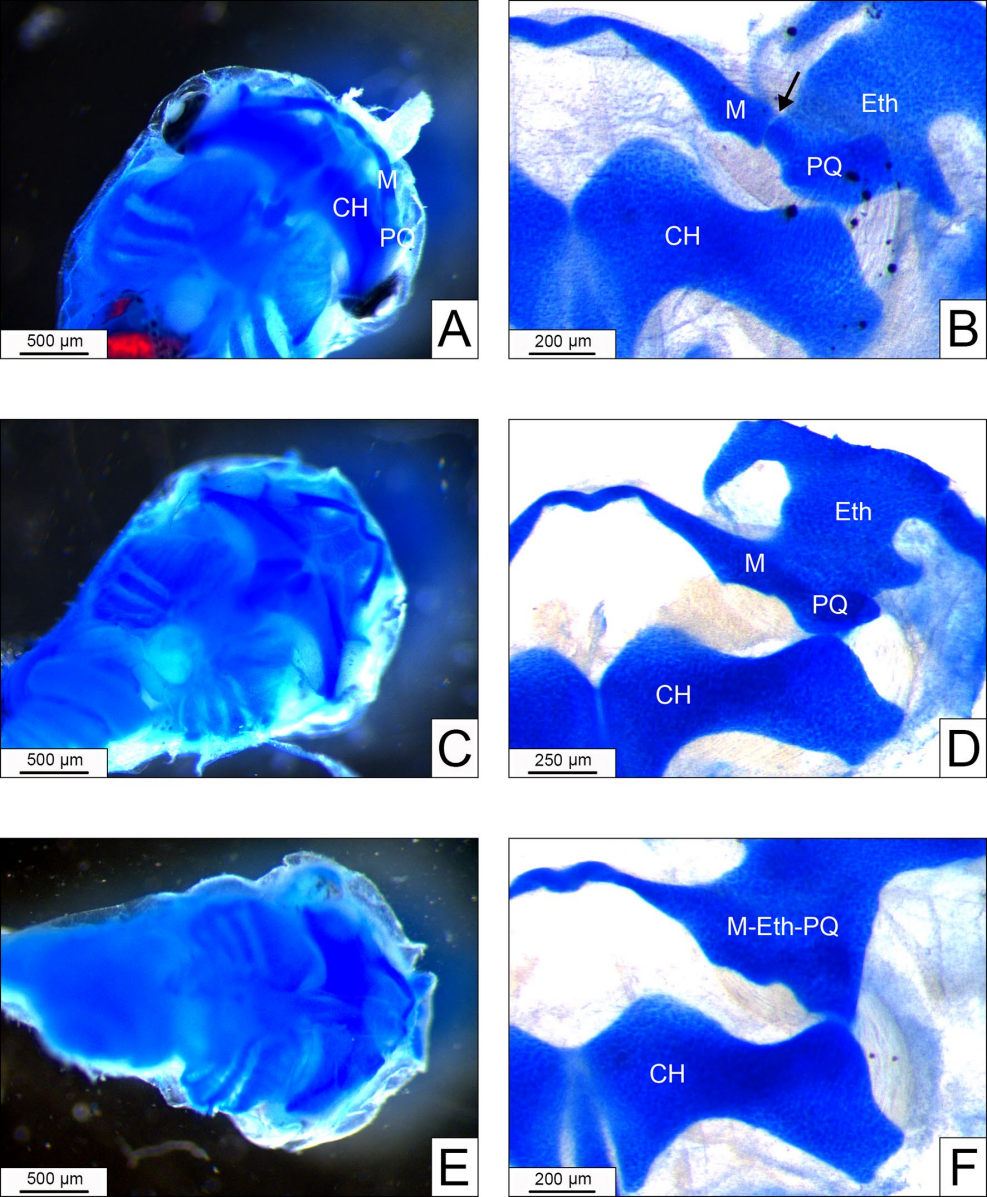
Appearance of whole mount **(A, C, E)** and flat mount **(B, D, F)** stained cartilages in a normal larva **(A, B)** and in larvae appearing as normal at the whole mount evaluation **(C, E)** showing moderate **(D)** or extended **(F)** fusions among ethmoidal (Eth), Meckel’s (M) and palatoquadrate (PQ) cartilages. In B note the articular space (arrow) between Meckel’s and palatoquadrate cartilages, not visible in the abnormal larvae **(D, F).** CH = ceratohyal cartilage

In detail, cartilages appeared fused (with a range from fusions visible only after flat mount to fused circular funnel-shaped cartilages also visible at the whole mount examination) (Fig. 3) with clear dose- and stage-related effects (Table 1). The overall NOAEL with R-FETAX approach was set at 3.9 μM.

**Fig. 4:**
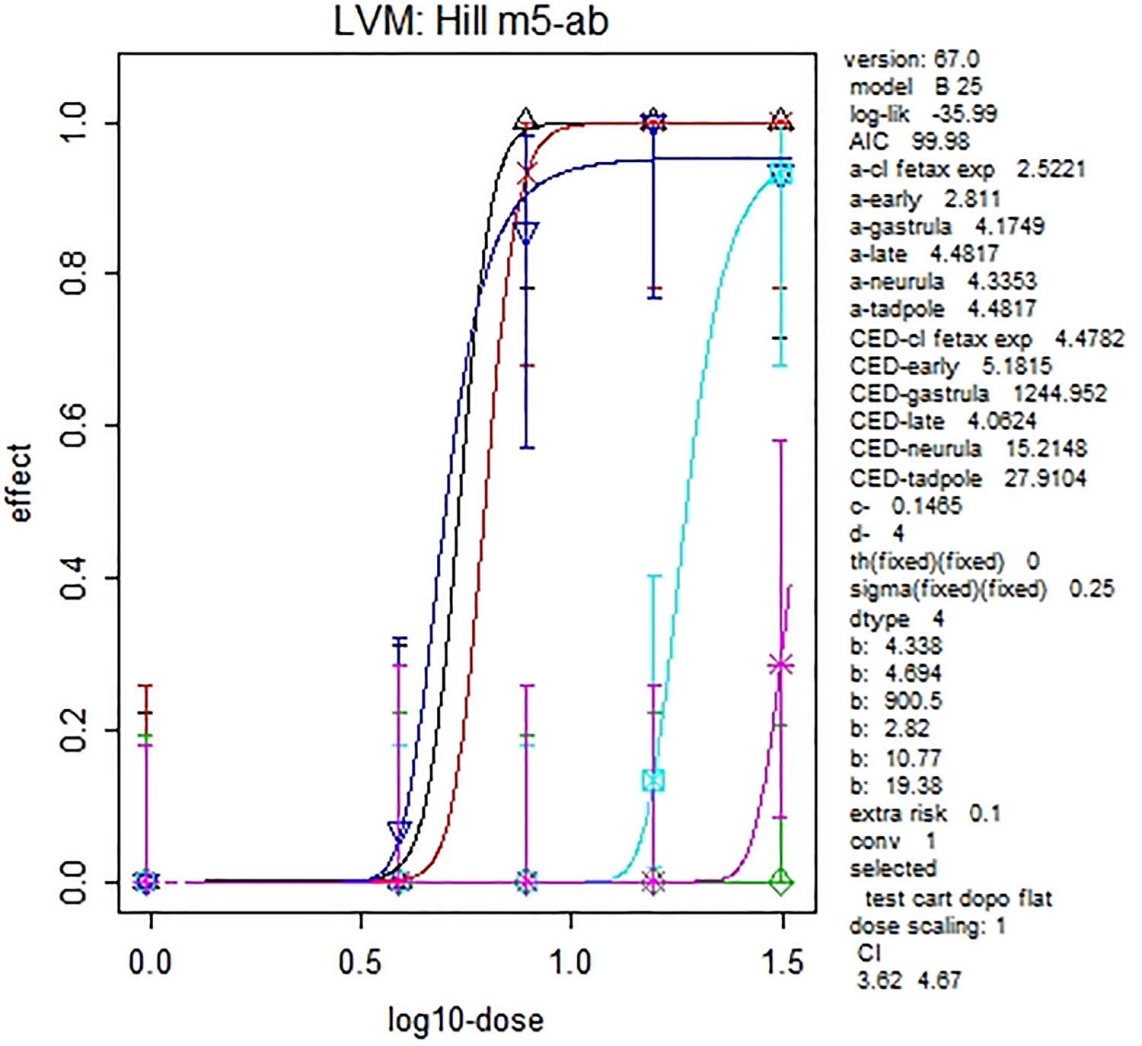
Hill model: dose–response curves obtained considering stage as covariate. The colors/symbols in the plot refer to the different exposure groups: black/upward triangle = classical FETAX exposure; green/diamond = gastrula; light blue/cross-square = neurula; red/ cross = early morphogenesis; dark blue/downward triangle = late morphogenesis; pink/cross-plus tadpole. Model evidenced late morphogenesis as the sensible subgroup

To evaluate stage-dependent embryo toxicity of FON, data were modeled by PROAST, and BMD confidence intervals (CIs) for BMR 10% were derived considering stage as covariate (Table  2; Fig. 4). Overlapping BMD CIs were obtained for whole test (classical FETAX exposure) and for early/late morphogenesis exposures showing similar sensitivity to FON.

**Table 2:** Benchmark doses (BMDs) for 10% benchmark responses (BMRs) with 95% confidence intervals (BMDL–BMDH) (μM) derived considering exposure stage as covariate

Exposure window	Exposure NF stages	BMD	BMDL	BMDH
Classical FETAX exposure	8–46	4.48	3.56	5.4
Gastrula	8–13	1244.95	31.5	Inf
Neurula	13–17	15.21	13.6	16.9
Early morphogenesis	13–26	5.18	4.07	5.83
Late morphogenesis	26–38	4.06	3.62	4.67
Tadpole	38–46	27.91	19.6	30.7

### Comparison of results obtained by different methods in the sensible subgroup (late morphogenesis window)

As the late morphogenetic period resulted in the sensible subgroup (Fig. 4), it was selected for further evaluations on results in different methods (external gross morphological evaluation of fresh samples, deglutition test, full cartilage evaluation including flat mount, whole mount cartilage evaluation, and M–PQ angle) (Table 3 and 4).

**Fig. 5:**
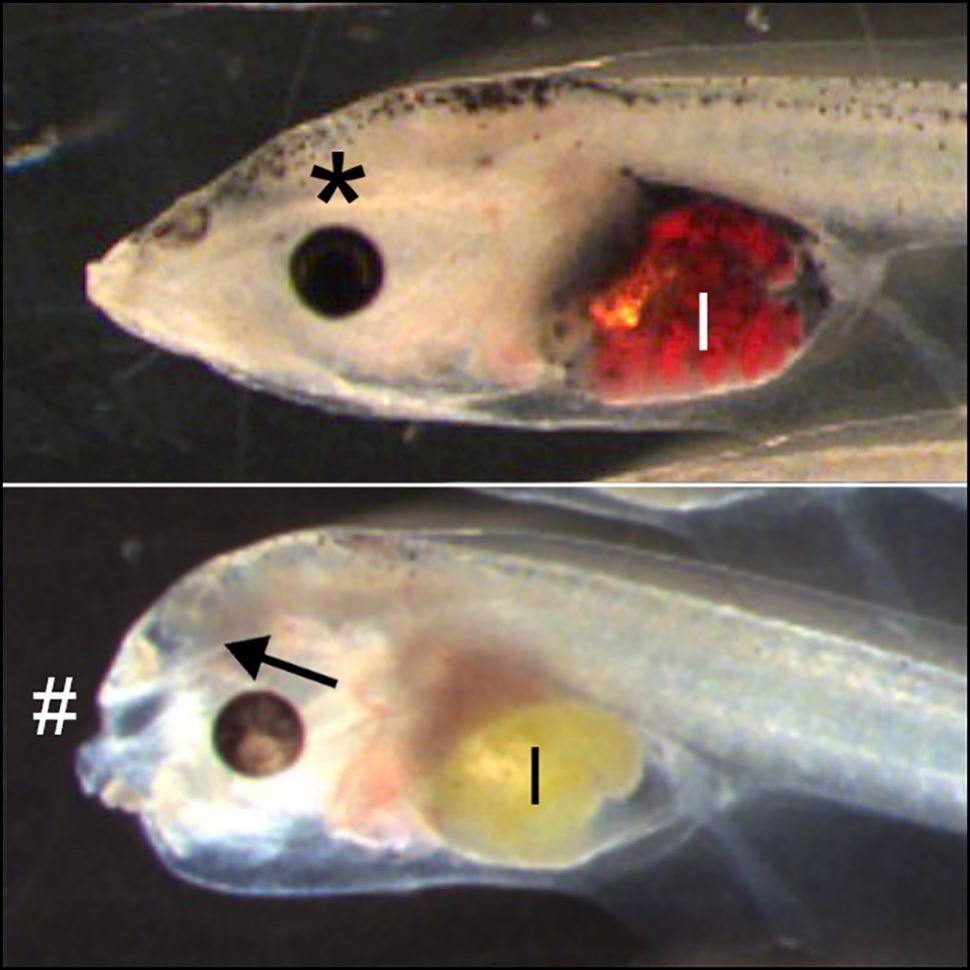
Lateral view of the anterior region of a normal larva (upper panel) with degl + phenotype and of an abnormal sample (lower panel) characterized by round head with bent encephalon (arrow), short snout (#) and degl− phenothype. I = intestine; * = normal linear encephalon

In detail, (i) the gross external evaluation showed some cases of abnormal anterior structures (round head: bent encephalon and short snout), with a dose-related trend (Table 3; Fig. 5); (ii) after 2 h of microplastic exposure (deglutition test), larvae displayed a degl− phenotype (absence of microplastics in the intestine) with a dose-related trend (Table 3; Fig. 2; Fig. 5); (iii) whole mount cartilage evaluation was able to detect abnormalities only at the highest concentration, while a dose-related effect was well evident with the full cartilage evaluation (Table 3; Fig. 3); (iv) the measurement of M–PQ angle (Fig. 2A) was not applicable in larvae with severe fusions (circular funnel-shaped cartilages) (Fig. 2B) and changes of the M–PQ angle were evident only at the FON highest dose group (Table 4).

**Table 3:** Percentage of abnormal larvae evaluated applying different morphological methods.

Method	Diagnosis	Fon concentration (μM)					*p*-value
		**0**	**3.9**	**7.8**	**15.6**	**31.2**	
Gross morphological evaluation	Abnormal	**0**	**0**	**0**	**7**	**20**	**0.011**
Deglutition test	Degl −	**7**	**13**	**40**	**100**	**87**	** < 0.0001**
Cartilage full evaluation	Fused (whole/flat mount)	**0**	**7**	**86**	**100**	**93**	** < 0.0001**
Cartilage whole mount evaluation	Abnormal	**0**	**0**	**0**	**0**	**6**	**0.0061**

Bold characters in the *p* column indicate significant values

Data recorded in the sensible subgroup (group exposed to FON at late morphogenetic stages). Statistics by Chi-square for trend calculated on frequencies

**Table 4:** M–PQ angle (M ± SD) measured in cartilage-stained larvae of the sensible subgroup (group exposed to FON at late morphogenetic stages).

Method	Fon concentration (μM)				
	**0**	**3.9**	**7.8**	**15.6**	**31.2**
					**
M–PQ angle (M ± SD)	94.1 ± 11.8	99.4 ± 8.4	105.6 ± 5.8	96.9 ± 10.4	64.5 ± 12.0

Bold ** indicate significant values

Statistics by ANOVA, followed by Tukey’s test. ** *p* < 0.0001 versus control

Full cartilage evaluation including flat mount, whole mount cartilage evaluation, external gross morphology, deglutition test, M–PQ angle data were singularly modeled to obtain dose–response curves and relative BMDs for BMR10%. Results showed only for deglutition test BMD central estimate and CI comparable to full cartilage values (Table5).

**Table 5:** Benchmark doses (BMDs) for 10% benchmark responses (BMRs) with 95% Confidence Intervals (BMDL– BMDH) (μM) calculated on data obtained by different method on late morphogenesis exposed group (sensible subgroup)

Exposure window	BMD	BMDL	BMDH
Gross morphological evaluation	17.34	9.69	inf
Deglutition test	5.52	2.92	6.89
Cartilage full evaluation	4.12	3.29	5.08
Cartilage whole mount evaluation	31.83	19.1	inf
M–PQ angle	21.7	17.5	22.5

### Comparison between full cartilage evaluation and deglutition test in the different exposure window groups

The evaluation of deglutition test predictivity for craniofacial defects was done by modeling and comparing data obtained by the two methods (full cartilage evaluation and deglutition test) in different developmental window groups. RPF calculation was performed after verification that log-likelihood test passed (*p* values > 0.05). Results showed thar the two assays were extremely similar in potency (Table 6).

**Table 6:** Relative potency factors (RPFs) with 95% confidence intervals (RPFL– RPFH) obtained in different exposed groups: deglutition test versus cartilage full evaluation (reference)

Exposure window	RPF	RPFL	RPFH
Classical FETAX exposure	1	0.57	1.76
Neurula	1.65	0.67	1.36
Early morphogenesis	0.97	0.89	1.05
Late morphogenesis	0.77	0.57	0.92
Tadpole	2.83	0.92	6.98

## Discussion

Both *X.* *laevis* and zebrafish models are considered suitable to evaluate craniofacial defects induced by a range of xenobiotics and biotics (Kennedy et al. 2017; Xu and Gye 2018; Staal et al. 2018; Battistoni et al. 2020; Heusinkveld et al. 2020; Zoupa et al. 2020; Huang et al. 2021), notwithstanding a predictive, simple, economic and, preservative test has not yet been suggested for routine evaluations.

The aim of the present work is the identification of a simple and reliable predictive method for craniofacial malformation in *X.* *Laevis* (R-FETAX). We used FON exposure in different developmental stages to obtain a wide spectrum of malformations with different severity grade (from punctiform fusions to funnel-shaped cartilages). Clear dose- and stage-related effects were detected using the full evaluation of cartilages, considered the gold standard method. The overall NOAEL with R-FETAX approach was set at 3.9 μM. Exposure during gastrulation was ineffective to induce any adverse effect at the tested concentrations (3.9–31.2 μM), while exposure during tadpole stages was effective only at the highest tested concentration. In early/late morphogenesis windows and the whole test (classical FETAX exposure), a clear dose relationship was observed from 7.8 μM or higher. The exposure during the neurula period was effective at concentrations reported in our works in the FON pathogenic pathway (Groppelli et al. 2005; Papis et al. 2007; Di Renzo et al. 2011b, e), with NOAEL fixed at 15.6 μM. Overall, the present results suggest R-FETAX windowed exposure to be a good approach to describe both dose- and stage-related effects of FON, with gastrula, early, and late morphogenesis and tadpole windows as the best representatives of different developmental phases.

In compliance with recent directions (Filipsson et al. 2003; Davis et al. 2011; EFSA Scientific Committee et al. 2017), we used the BMD approach. This is more advantageous than NOAEL, fitting experimental data to mathematical models to derive BMD relative 95% CIs for fixed BMRs. According to EFSA Scientific Committee (2017) BMDL10s (lower confidence bounds of BMDs for 10% response level) is suggested for risk assessment purposes.

In the present study, the comparison among CIs of BMD for BMR 10% allowed to determine the sensitivity to FON of the different developmental phases: the exposure during the whole test (classical FETAX exposure) and during the early and late morphogenetic periods resulted in a similar response; consequently, these developmental phases seem the most sensitive ones, while neurulation and tadpole period was one order of magnitude less sensitive.

Due to the narrower BMD CI, late morphogenetic period resulted as the sensible subgroup for modeling. For this reason, the evaluation of different methods to predict craniofacial defects was done only on data obtained in the group exposed during the late morphogenetic window. Data obtained applying each method were modeled and BMD CIs for BMR10% derived. The full evaluation of cartilages is confirmed as the gold standard in *X.* *laevis*, with lower absolute BMD value and narrow CI. Among the disadvantages of this method, time and reagent consumption is a concern. Moreover, the main criticisms are the enormous time spent by extremely skilled personnel in the application of the flat mount technique and the impossibility of using samples for further different morphological or molecular investigations. The evaluation of cartilages limited to the whole mount assay and the alternative technique applied on photos (M–PQ angle) failed to describe the complete picture and appeared one order of magnitude less sensitive. The measure of the mandible angle, proposed in zebrafish to evaluate craniofacial abnormalities (Zoupa et al. 2020), is a novelty in *X.* *laevis*. This method, based on cartilage staining, shows the same criticisms (time-and reagent-consuming, excluding further evaluations on the same specimens). Gross external morphology evaluation on fresh samples allows the preservation of samples, but was insufficient to evaluate craniofacial defects. By contrast, deglutition test results were very close to those obtained by the gold standard method, as confirmed by RPF values resulting in extremely similar potency of deglutition test versus the gold standard method.

In conclusion, we consider deglutition test the most predictive and reliable of severe alteration on craniofacial development. This is a rapid, unexpensive test, and the evaluation of positive/negative deglutition can be done by base-trained personnel. Most important, it is a vital test and allows to describe complex syndromic pictures using further techniques on the same samples.

We suggest the deglutition test in routine *X.* *laevis* evaluation and propose this rapid and sensitive method also in other alternative models like zebrafish.
